# Light-induced in situ reconstruction of CoOOH-modified TiO_2_/CoNi-LDH heterojunction photoanode: achieving excellent photoelectrochemical cathodic protection and bacterial inactivation

**DOI:** 10.1038/s41377-026-02328-z

**Published:** 2026-05-11

**Authors:** Meiqi Wang, Yiqing Tang, Juan Liu, Xinyue Feng, Zheng Kuang, Yingnan Qin, Jing Tian, Ning Wang, Jing Wang

**Affiliations:** 1 College of Chemical Engineering, Qingdao University of Science and Technology, Qingdao, China; 2 State Key Laboratory of Advanced Optical Polymer and Manufacturing Technology, Qingdao University of Science and Technology, Qingdao, China

**Keywords:** Nanoparticles, Solar energy and photovoltaic technology, Photonic devices

## Abstract

Corrosion and biofouling of metals in marine environments are critical issues affecting the long-term stability of marine engineering infrastructure. Traditional protection methods suffer from limitations such as high energy consumption and environmental pollution. Photoelectrochemical cathodic protection (PECCP) technology utilizes solar energy to drive the transfer of photogenerated electrons from semiconductors to metal surfaces, enabling green and low-energy-consumption corrosion protection. However, its core challenge lies in developing efficient and stable photoelectrode materials. In this study, a TiO_2_/CoNi-LDH composite photoanode was fabricated via hydrothermal and electrodeposition methods. It was found that under illumination, CoNi-LDH undergoes in situ reconstruction to generate CoOOH as a cocatalyst. The composite material exhibited excellent photoelectrochemical cathodic protection performance in a simulated seawater environment, providing a potential shift of 380 mV for coupled 304 stainless steel under intermittent illumination. Simultaneously, it demonstrated high antibacterial efficiency, achieving a 100% inactivation rate against *Pseudomonas aeruginosa* within 120 min. Structural characterization and theoretical calculations revealed that the in situ formation of CoOOH enhances interfacial charge transfer and promotes the generation of reactive oxygen species, thereby synergistically improving the anti-corrosion and anti-biofouling performance of the material. This study provides a novel strategy for developing integrated marine protective materials with long-term corrosion resistance and biofouling prevention capabilities.

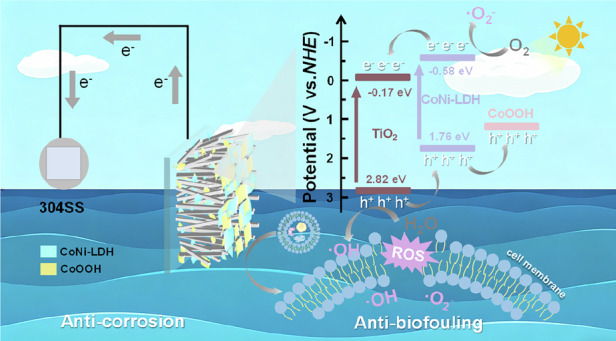

## Abstract (web summary)

In situ reconstruction of CoNi-LDH generates CoOOH, enhancing charge transfer and ROS generation for photoelectrochemical cathodic protection and anti-biofouling.

## Introduction

Metal corrosion is a common phenomenon in nature, fundamentally arising from electrochemical reactions between metals and their surrounding environment (e.g., oxygen, water, electrolytes), which lead to structural damage and performance degradation of metallic materials^1^. Oceans cover approximately 70% of the Earth’s surface, and the high salinity, high humidity, and complex biochemical conditions of seawater result in a corrosion rate that is significantly higher than in terrestrial environments. This poses serious threats to infrastructure such as ships, offshore platforms, and subsea pipelines^2–4^. Current primary anti-corrosion technologies include coating application, corrosion inhibitor addition, sacrificial anode protection, and impressed current cathodic protection^5–9^. However, conventional protection methods are often associated with high energy consumption, environmental pollution, and substantial demands on human and material resources. For instance, sacrificial anode protection requires periodic replacement of anode materials, while impressed current protection continuously consumes electrical energy. Moreover, volatile organic compounds present in organic coatings can have adverse effects on the ecological environment.

As an emerging anti-corrosion technology, photoelectrochemical cathodic protection (PECCP) operates by using solar energy to drive the transfer of electrons from a semiconductor to the metal surface, thereby maintaining an electron-rich state on the metal and achieving cathodic protection^10–15^. This approach is not only environmentally friendly but also significantly reduces energy consumption, showing promising application prospects. To optimize the performance of photoelectrodes, researchers have conducted extensive investigations. For example, Xu et al. constructed a TiO_2_/CdS@ZnS heterojunction using the successive ionic layer adsorption and reaction method, successfully polarizing the coupling potential of 304 stainless steel (304 SS) to −1.05 V (vs. SCE) and significantly enhancing the interfacial polarization level^16^. Meanwhile, Tian et al., through Mo-doped WO_3_/CdZnS systems, not only achieved a photogenerated potential drop of 323.6 mV but also extended the effective protection of PECCP to dark conditions without illumination, leveraging the excellent charge storage properties of the material^17^. Although the aforementioned studies have made progress in terms of charge separation and energy storage, the design and construction of photoelectric conversion materials that combine broad-spectrum response, long-term chemical stability, and multifunctionality (such as combined antibacterial and anticorrosion properties) under complex service environments remain critical challenges to be addressed in the field of PECCP.

TiO_2_, as an excellent n-type semiconductor material, possesses stable electron transport capabilities and good chemical stability. However, its wide bandgap (approximately 3.2 eV) restricts light absorption to the ultraviolet region, which constitutes only about 5% of the solar spectrum, resulting in low utilization efficiency of sunlight and limiting its broader application^18–20^. Layered double hydroxides (LDHs), a class of anionic materials with unique layered structures, offer tunable compositions enabling broad-spectrum light absorption. Due to their relatively narrow band gaps, LDHs have been widely investigated for applications such as photoelectrochemical water splitting and photoelectrochemical cathodic protection^21–24^, including materials like ZnCo-LDH^25^, NiFe-LDH^26^, and CoNi-LDH^27^.

Although compositing LDHs with TiO_2_ generally enhances the photoelectrochemical cathodic protection performance of the resulting material, the interface typically forms a Type-II heterojunction. In this configuration, both photogenerated electrons and holes transfer from higher to lower energy levels. While this effectively improves the separation efficiency of the charge carriers, it concurrently reduces their redox potentials, which is detrimental for applications requiring high redox power, such as anti-corrosion and anti-biofouling^28^. Modifying TiO_2_ with co-catalysts can lower the reaction overpotential and accelerate reaction kinetics. For instance, Reyes-Ahmad et al.^29^ constructed a dual co-catalyst system by depositing gold Au and Pt nanoparticles onto the TiO_2_ surface via a co-deposition method, which demonstrated superior charge transfer capability and high conductivity.

Recent studies have shown that some transition metal oxides can spontaneously undergo hydroxylation during electrochemical processes, forming metal oxyhydroxides (e.g., CoOOH, NiOOH) as the true active sites, thereby reducing the reaction energy barrier and enhancing kinetics. For example, Chen et al. proposed a spin polarization engineering strategy based on manganese ion doping. By modulating the electronic structure, the reaction energy barrier for oxidizing Co^2+^ to Co^3+^ was significantly reduced, thereby efficiently promoting the formation of the active intermediate CoOOH. This work opens up a new pathway for driving the dynamic reconstruction of active (oxy)hydroxides through electronic state regulation^30^. Wang et al.^31^ constructed a self-supported electrode by hydrothermally growing CoMoO_4_ nanosheets on Cu(OH)_2_ nanorod-covered copper foam. During the hydrogen evolution reaction (HER), Co^2+^ in CoMoO_4_ interacted with OH^−^ ions from the electrolyte, spontaneously reconstructing into molybdenum-doped CoOOH nanosheets as the active phase. This in situ formation of metal oxyhydroxides provides a conceptual foundation for the present study.

In this study, a TiO_2_/CoNi-LDH heterojunction photoanode was constructed on a fluorine-doped tin oxide (FTO) substrate via a combination of hydrothermal synthesis and electrodeposition. Unlike conventional static heterojunction interfaces, this work revealed a significant in situ dynamic reconstruction behavior in the system upon photoexcitation. A visible color change of the electrode from bluish-green to brownish-yellow was observed. Combined with XRD and Raman spectroscopic characterization, this change confirmed the hydroxylation-driven transformation of Co(OH)_2_ into the strongly oxidative active phase CoOOH, facilitated by photogenerated holes.

This in situ formed CoOOH co-catalyst not only fundamentally optimizes the interfacial charge separation kinetics, but also, through the synergistic effect of its high-valence cobalt species and photogenerated free radicals, overcomes the longstanding limitation of low inactivation efficiency against *Pseudomonas aeruginosa* by traditional photocatalytic materials. The results demonstrate that under intermittent illumination, the composite material induces a polarization shift of 380 mV in the potential of 304 SS and achieves 100% inactivation of *Pseudomonas aeruginosa* within 120 min. This discovery not only clarifies the structural evolution mechanism of transition metal hydroxides under photoexcitation but also provides a novel strategy for designing stable photoanodes with dual antibacterial and anticorrosion functionalities.

## Results

**Fig. 1:**
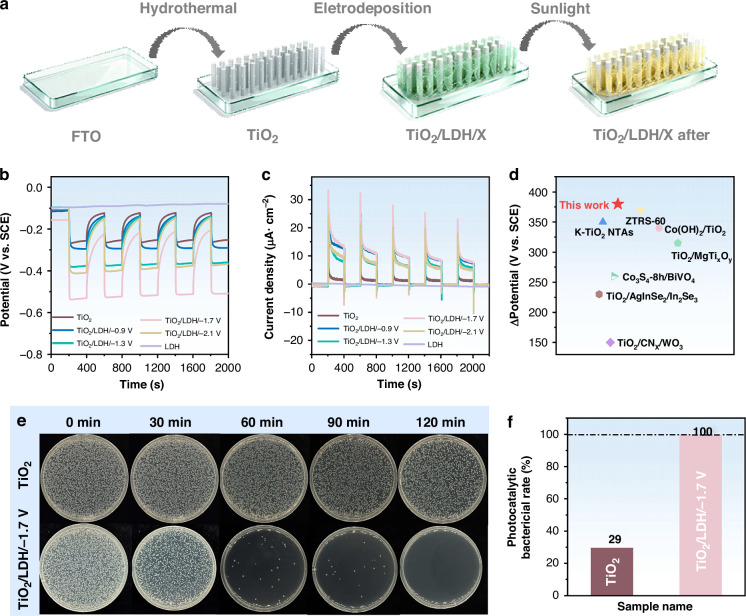
Preparation, photoelectrochemical performance, and antibacterial activity of TiO_2_/CoNi-LDH composites. **a** Schematic diagram illustrating the preparation process of the TiO_2_/CoNi-LDH composite, **b** photopotential response and **c** photocurrent density variation of fabricated TiO_2_, CoNi-LDH, and a series of TiO_2_/CoNi-LDH composite photoelectrodes coupled with 304 SS under intermittent simulated solar illumination, **d** comparison with previously reported materials, **e** images of colony formation plates and **f** corresponding histogram of bactericidal rates for *Pseudomonas aeruginosa* after different sample treatments under varying illumination durations (120 min)

To assess the potential for practical application in marine environments, the fabricated TiO_2_, CoNi-LDH, and a series of TiO_2_/CoNi-LDH samples were immersed in a 3.5 wt% NaCl solution. As shown in Fig. 1b, coupling TiO_2_ with 304 SS resulted in a photo-induced potential drop of 150 mV. In contrast, the potential of CoNi-LDH coupled with 304 SS showed negligible change upon illumination, indicating that pure CoNi-LDH cannot provide cathodic protection for 304 SS. This limitation arises because its relatively narrow bandgap impedes efficient separation of photogenerated electrons and holes. After depositing CoNi-LDH onto TiO_2_, the TiO_2_/CoNi-LDH composite photoelectrodes fabricated at different deposition potentials exhibited significantly higher photo-induced potential drops compared to pure TiO_2_ or pure CoNi-LDH electrodes. Notably, the potential drop initially increased and then decreased as the deposition potential became more negative. This trend is likely attributable to insufficiently negative potentials causing non-uniform deposition of CoNi-LDH nanosheets, leading to discontinuous charge transport pathways. Conversely, excessively negative potentials promote rapid nucleation, forming thick and compact layered structures that cover the underlying TiO_2_ layer and prevent light absorption. Analysis of Fig. 1b identifies an optimal deposition potential of −1.7 V for TiO_2_/CoNi-LDH, yielding a potential drop of approximately 380 mV. Figure 1c, which compares the influence of different deposition potentials on the TiO_2_/CoNi-LDH composite photoelectrodes, reveals that the variation in photocurrent density follows a similar trend to that of the potential drop. The TiO_2_/CoNi-LDH/−1.7 V electrode exhibited the highest photocurrent density (~14 µA cm^−2^), signifying its superior photogenerated carrier separation efficiency and its capacity to supply more photogenerated electrons for cathodic protection of 304 SS. Based on a comparison of recent literature data (Fig. 1d)^32–38^, this composite material demonstrates a superior potential drop compared to other materials, reflecting its excellent photogenerated cathodic protection performance. Figures S1 and S2 show optical microscopy images of 304 SS coupled with the TiO_2_/CoNi-LDH/−1.7 V composite photoanode before and after immersion in a 3.5 wt% NaCl solution for 24 h. Figure S1 reveals that the surface of the 304 SS coupled with the composite photoanode remains smooth, with no signs of corrosion observed. In contrast, Fig. S2 presents optical microscopy images of bare 304 SS under the same conditions (3.5 wt% NaCl solution, 24 h). A clear comparison shows that the unprotected 304 SS surface exhibits obvious corrosion.

To evaluate the *Pseudomonas aeruginosa* inactivation performance of TiO_2_ and the TiO_2_/CoNi-LDH/−1.7 V composite photoelectrode, bactericidal experiments were conducted under simulated solar illumination for varying durations. The resulting bactericidal efficacy is presented in Fig. 1e. The results demonstrate that both TiO_2_/CoNi-LDH/−1.7 V and TiO_2_ exhibit discernible bactericidal activity, with the composite photoelectrode showing particularly pronounced efficacy. Figure S3 presents the blank control under illumination only. After 120 min of light exposure, no significant reduction in colony count was observed, indicating that light alone does not affect the growth of *Pseudomonas aeruginosa*. Figure 1f shows the sterilization rates of different materials after 120 min of illumination. The sterilization rate of TiO_2_ was only 29%, whereas the TiO_2_/CoNi-LDH/−1.7 V composite achieved a sterilization rate of 100%. This indicates that the deposition of CoNi-LDH significantly enhances the performance of TiO_2_ in inactivating *Pseudomonas aeruginosa*^14^.

**Fig. 2:**
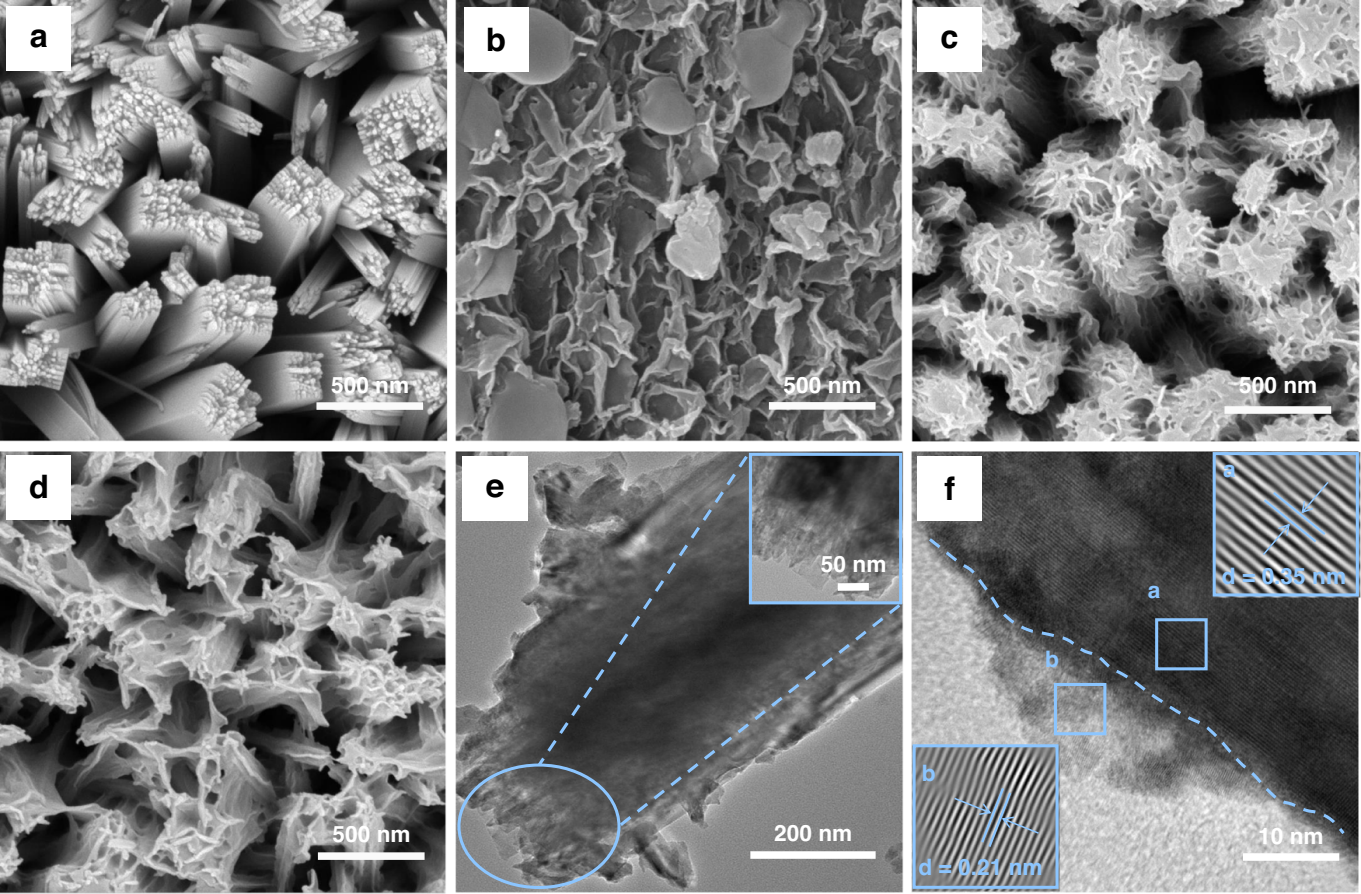
Morphological characterization of TiO2, CoNi-LDH, and TiO_2_/CoNi-LDH composites. SEM images of **a** TiO_2_, **b** CoNi-LDH, **c** TiO_2_/CoNi-LDH/−1.7 V, and **d** TiO_2_/CoNi-LDH/−1.7 V post-treatment, **e** TEM and **f** HRTEM images of the TiO_2_/CoNi-LDH/−1.7 V composite

Figure 2a–d presents the SEM images of the series of fabricated photoanodes. After the hydrothermal reaction, TiO_2_ nanorods vertically aligned on the FTO substrate (Fig. 2a). CoNi-LDH directly electrodeposited on the FTO surface without a TiO_2_ nanorod underlayer exhibited agglomerated nanosheets with irregular sizes and non-uniform dispersion (Fig. 2b). As shown in Fig. 2c, the sample prepared on the TiO_2_ substrate at a deposition voltage of −1.7 V (TiO_2_/CoNi-LDH/−1.7 V) demonstrates a uniform coating of CoNi-LDH, forming a three-dimensional network composed of interconnected, irregular nanosheets on the TiO_2_ nanorods. These CoNi-LDH nanosheets possess a larger specific surface area, which is anticipated to provide more active sites for the photoelectrochemical cathodic protection and bactericidal processes. The EDS results (Fig. S4) confirm the presence of elements such as Ti, O, Co, and Ni, while the element mapping results (Fig. S5) demonstrate the uniform distribution of these elements across the surface of the TiO_2_/CoNi-LDH/−1.7 V electrode. The SEM image of the TiO_2_/CoNi-LDH/−1.7 V sample after the photoelectrochemical cathodic protection test (Fig. 2d) reveals that the nanosheets increased in size and formed a sparser network compared to the pristine sample. This morphological change is likely attributable to the light-induced reconstruction of CoNi-LDH, leading to the branching of the nanosheet structure.

Figure 2e presents a low-magnification TEM image of the TiO_2_/CoNi-LDH/−1.7 V sample. The high-resolution TEM (HRTEM) image (Fig. 2f) reveals distinct lattice fringes and the heterojunction interface. The measured lattice spacings of 0.35 nm and 0.21 nm correspond to the (101) crystal plane of TiO_2_ and the (107) plane of CoNi-LDH, respectively. This provides clear evidence for the successful construction of the TiO_2_/CoNi-LDH heterojunction.

**Fig. 3:**
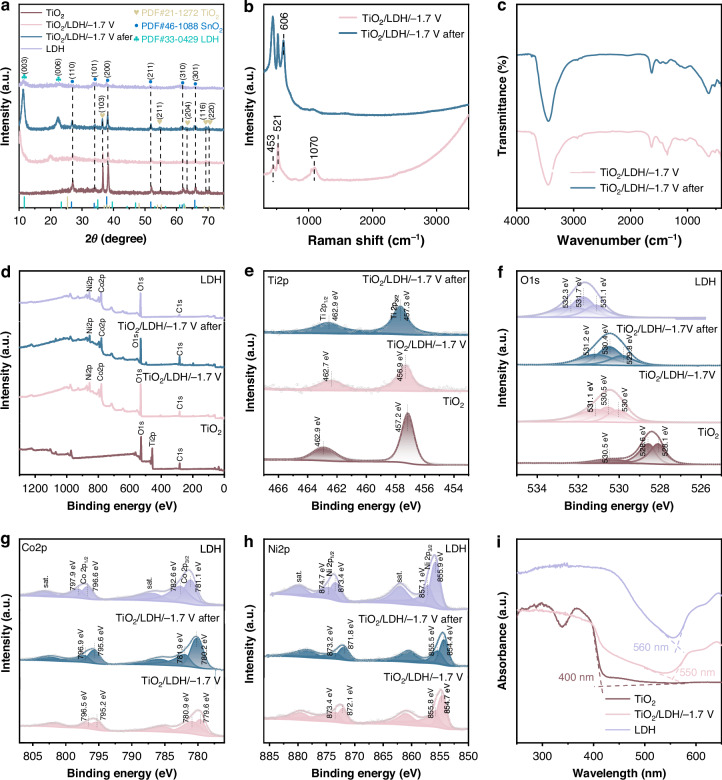
Structural and optical characterization of the series of photoelectrodes. **a** XRD patterns of the series of photoelectrodes, **b** Raman spectra and **c** FT-IR spectra of TiO_2_/CoNi-LDH/−1.7 V and TiO_2_/CoNi-LDH/−1.7 V after testing, **d**–**h** XPS spectra of the series of photoelectrodes, **i** UV-Vis absorption spectra of the series of photoelectrodes

Figure 3a shows the XRD patterns of the series of photoanodes. The diffraction peaks corresponding to SnO_2_ (PDF #46-1088) from the FTO substrate are observed in all four samples: TiO_2_, TiO_2_/CoNi-LDH/−1.7 V, TiO_2_/CoNi-LDH/−1.7 V after, and CoNi-LDH. In the XRD pattern of the TiO_2_ sample, the diffraction peaks at 36.94°, 55.06°, and 70.31° are indexed to the (103), (211), and (220) crystal planes of the anatase phase (PDF #21-2176), respectively. For the CoNi-LDH sample, the characteristic peaks located at 11.57° and 23.34° are assigned to the (003) and (006) planes of cobalt-nickel layered double hydroxide (PDF #33-0429), confirming its successful synthesis. All diffraction peaks are sharp, and no impurity peaks are detected, indicating high crystallinity and phase purity of the as-prepared materials.

The XRD pattern of the TiO_2_/CoNi-LDH/−1.7 V composite exhibits characteristic peaks of both TiO_2_ and CoNi-LDH. However, compared to the CoNi-LDH grown directly on FTO, the diffraction peaks of CoNi-LDH in the composite show a slight shift towards lower angles. This shift can be attributed to the refinement of CoNi-LDH nanosheet crystallites grown on the TiO_2_ nanorods. Notably, in the pattern of the TiO_2_/CoNi-LDH/−1.7 V after sample (post-testing), the CoNi-LDH diffraction peaks shift towards higher angles compared to the pristine composite, accompanied by a further increase in peak intensity. This change is likely induced by the light-driven reconstruction of the CoNi-LDH structure^39^.

To further elucidate the reconstruction mechanism of the TiO_2_/CoNi-LDH/−1.7 V after sample, Raman and FT-IR spectroscopy analyses were conducted, with the results shown in Fig. 3b, c, respectively. In the Raman spectrum (Fig. 3b), the band located at 521 cm^−1^ can be attributed to the symmetric stretching mode (Ag) of Co−O. The peak at 453 cm^−1^ is derived from the O−Co−O bending mode^40^. The peak at 1070 cm^−1^ corresponds to the vibration of CO_3_^2−^ in the interlayer space of the LDH. This, together with the broad peak observed at 1355 cm^−1^ in the FT-IR spectrum, indicates that the material retains the characteristic features of a typical layered double hydroxide^41^. After light treatment of the composite material, the enriched reactive oxygen species in the interlayer space oxidize the CO_3_^2−^ to CO_2_ or other carbon products, leading to the removal of the interlayer carbonate ions and the disappearance of the corresponding characteristic peaks. Due to changes in the interlayer spacing and lattice strain induced by anion removal, the original peaks at 521 cm^−1^ and 453 cm^−1^ shift slightly toward lower wavenumbers. Meanwhile, a new characteristic peak emerges at 606 cm^−1^, which is assigned to CoOOH^42^.

XPS was employed to analyze the surface elemental composition and valence states of the samples, with the results presented in Fig. 3d–h. The survey spectra in Fig. 3d confirm the presence of O, Co, Ti, and Ni elements. Figure 3e–h displays the high-resolution spectra along with peak deconvolution results for the Ti 2p, O 1s, Co 2p, and Ni 2p orbitals, respectively. From the high-resolution Ti 2p spectra of TiO_2_, TiO_2_/CoNi-LDH/−1.7 V, and TiO_2_/CoNi-LDH/−1.7 V after testing, shown in Fig. 3e, it can be observed that TiO_2_ exhibits two distinct peaks at 457.2 eV and 462.9 eV, corresponding to the Ti 2p_3/2_ and Ti 2p_1/2_ orbitals, respectively. After loading CoNi-LDH, the binding energies for the TiO_2_/CoNi-LDH/−1.7 V photoanode shift to 457.3 eV and 463.0 eV. This shift is attributed to electron transfer from TiO_2_ to CoNi-LDH upon contact, resulting in a decrease in electron cloud density around Ti and an increase in the effective nuclear charge experienced by the inner-shell electrons^43,44^. Furthermore, after electrochemical testing, the peak positions of TiO_2_/CoNi-LDH/−1.7 V shifted slightly toward higher binding energies compared to TiO_2_/CoNi-LDH/−1.7 V. This indicates that after the in situ conversion of CoNi-LDH into CoOOH on TiO_2_/CoNi-LDH, electrons are transferred from TiO_2_/CoNi-LDH to CoOOH. As shown in the XPS spectra of Co 2p (Fig. 3g) and Ni 2p (Fig. 3h), the binding energies of both Co and Ni in the LDH shift toward lower values compared to those in TiO_2_/CoNi-LDH/−1.7 V. This further confirms that upon contact, electrons are transferred from TiO_2_ to CoNi-LDH. As a result, CoNi-LDH gains electrons, leading to an increased electron cloud density around the Co and Ni atoms, which reduces the effective nuclear charge experienced by their inner-shell electrons. This observation is consistent with the results obtained from the Ti 2p spectra. Furthermore, in the O 1 s results shown in Fig. 3f, the binding energies located at 528.1 eV, 528.6 eV and 530.5 eV correspond to the lattice oxygen, adsorbed oxygen, and adsorbed water of TiO_2_, respectively, while the binding energies at 531.1 eV, 531.7 eV and 532.3 eV correspond to the lattice oxygen, adsorbed oxygen, and adsorbed water of CoNi-LDH, respectively. Compared to TiO_2_ and CoNi-LDH, the binding energies of lattice oxygen, adsorbed oxygen, and adsorbed water in TiO_2_/CoNi-LDH/−1.7 V and TiO_2_/CoNi-LDH/−1.7 V after testing shift toward higher and lower binding energies, respectively. This also indicates that the construction of the heterojunction induces charge transfer between the two materials.

Based on the comprehensive XPS results, it can be inferred that due to the difference in Fermi levels between TiO_2_ and CoNi-LDH, electron transfer occurs from TiO_2_ to CoNi-LDH upon their contact. This leads to the formation of an electron-rich region at the CoNi-LDH interface and an electron-deficient region at the TiO_2_ interface, consequently establishing a heterojunction electric field pointing from TiO_2_ toward CoNi-LDH. Similarly, owing to the difference in Fermi levels between CoOOH and TiO_2_/CoNi-LDH, when TiO_2_/CoNi-LDH comes into contact with CoOOH, an electron-rich region forms at the CoOOH interface, while an electron-deficient region develops at the TiO_2_/CoNi-LDH interface. This results in the formation of a heterojunction electric field directed from TiO_2_/CoNi-LDH toward CoOOH.

The optical absorption range of the composite materials was analyzed using UV-vis diffuse reflectance spectroscopy. As shown in Fig. 3i, the absorption edges of TiO_2_ and CoNi-LDH are positioned at 400 nm and 560 nm, respectively. The TiO_2_/CoNi-LDH/−1.7 V composite exhibits an absorption edge at 550 nm, demonstrating a distinct red shift relative to pure TiO_2_ and a slight blue shift compared to pristine CoNi-LDH. This phenomenon is attributed to the successful construction of a TiO_2_/CoNi-LDH heterojunction, which effectively broadens the light-harvesting range of TiO_2_ and extends the photo-responsive region of the composite.

**Fig. 4:**
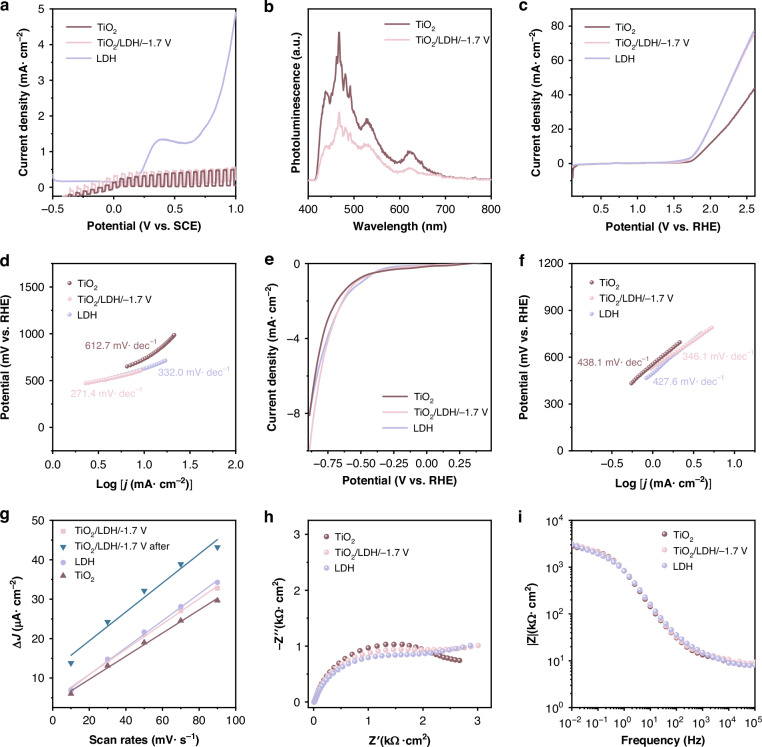
Photoelectrochemical and electrocatalytic performance of the series of photoelectrodes. **a** LSV curves for photoresponse and **b** PL spectra of the series of photoelectrodes, **c** OER polarization curves and **d** the corresponding Tafel slopes of the series of photoelectrodes, **e** HER polarization curves and **f** the corresponding Tafel slopes of the series of photoelectrodes, **g** current density variation of the series of photoelectrodes measured by CV at different scan rates, **h** EIS and **i** Bode curves of photoelectrodes

To further validate the photoelectrochemical performance of the photoelectrode series under intermittent simulated solar illumination, photoinduced LSV measurements were conducted in 3.5 wt% NaCl solution. The resulting current density versus applied potential profiles are presented in Fig. 4a. CoNi-LDH exhibits no photocurrent response across the entire scanning range, while TiO_2_ and TiO_2_/CoNi-LDH/−1.7 V show varying degrees of increase in photocurrent density with increasing bias voltage. This is attributed to the voltage‑driven facilitation of photogenerated charge carrier separation. Moreover, TiO_2_/CoNi-LDH/−1.7 V demonstrates the highest photocurrent throughout the entire scanning range, indicating its optimal photogenerated charge carrier separation efficiency. PL spectra of TiO_2_ and TiO_2_/CoNi-LDH/−1.7 V are compared in Fig. 4b. The TiO_2_ photoelectrode displays a pronounced PL emission peak, whereas the TiO_2_/CoNi-LDH/−1.7 V composite exhibits markedly attenuated peak intensity. This quenching effect originates from the formation of well-constructed heterojunction interfaces between the components, which facilitates efficient separation of photogenerated carriers. Consequently, a greater quantity of photogenerated electrons can be injected into the 304 SS substrate, enabling enhanced cathodic protection.

Figure 4c–f characterizes the electrochemical performance of the photoanode under different polarization conditions, directly revealing the intrinsic relationship between the material’s microstructure and its dual-functional protective efficacy. In the positive potential polarization region (Fig. 4c, d), the evaluation of water oxidation (OER) kinetics shows that compared to pure TiO_2_, the TiO_2_/CoNi-LDH/−1.7 V composite exhibits the lowest Tafel slope (271.4 mV/dec^−1^). This indicates that the introduction of LDH significantly accelerates the migration and consumption of photogenerated holes. The enhanced ability of these holes to participate in oxidation reactions forms the physicochemical basis for generating high concentrations of reactive oxygen species, providing an electrochemical explanation for the material’s excellent antibacterial performance.

Conversely, in the negative potential polarization region (Fig. 4e, f), the TiO_2_/CoNi-LDH/−1.7 V composite demonstrates a lower overpotential and a smaller Tafel slope. This proves that under cathodic polarization conditions, this heterostructure can more effectively transport photogenerated electrons to the surface of the coupled metal. This sustained electron injection ensures a stable negative shift in the potential of the metal substrate, thereby providing durable kinetic support for enhanced photoelectrochemical cathodic protection and effectively inhibiting the corrosion process.

The electrochemically active surface area (ECSA) is a crucial parameter for evaluating the performance of materials in photoelectrochemical cathodic protection. It can be determined by measuring the double-layer capacitance (*C*_dl_), which is obtained from the slope of the linear relationship between the charging current density and the scan rate in CV tests. A larger slope indicates a greater *C*_dl_ and, consequently, a larger ECSA, signifying more active sites available for electrochemical reactions. Figure S6 presents the CV curves of TiO_2_, CoNi-LDH, TiO_2_/CoNi-LDH/−1.7 V, and the TiO_2_/CoNi-LDH/−1.7 V sample after light irradiation at different scan rates. The corresponding comparison of the electrochemical active surface area (ECSA) results is shown in Fig. 4g. Compared to pure TiO_2_, the TiO_2_/CoNi-LDH/−1.7 V composite exhibits a significantly increased slope, indicating that the introduction of CoNi-LDH effectively mitigates the inherent limitation of insufficient active sites in TiO_2_ itself. The slope of the composite material reaches its maximum value after light treatment. This is attributed to the light-induced formation of the CoOOH co-catalyst, which establishes a density of active sites on the electrode surface substantially higher than that of conventional materials. This constitutes the reactive foundation for the dual-functional protection: regarding the antibacterial performance, the abundant active sites greatly promote the trapping of photogenerated holes and surface redox reactions, thereby efficiently generating reactive oxygen species; regarding the anti-corrosion performance, these sites provide numerous “transport channels” for the transfer of photogenerated electrons to the 304 SS, contributing to more efficient and longer-lasting cathodic polarization protection.

Figure 4h displays the Nyquist plots. The diameter of the semicircular arc in the high-frequency region reflects the charge transfer resistance, where a smaller arc radius indicates lower resistance and more efficient charge carrier migration. Pure TiO_2_ shows the largest arc radius, while after modification with CoNi-LDH, the TiO_2_/CoNi-LDH/−1.7 V composite photoanode exhibits a significantly reduced arc radius, confirming that CoNi-LDH effectively enhances the otherwise poor charge transfer capability of TiO_2_. The lower charge transfer resistance implies that photogenerated electrons can be injected into the 304 SS substrate more smoothly and rapidly, thereby driving more efficient cathodic polarization for corrosion protection. Simultaneously, photogenerated holes can also migrate to the material surface more swiftly to participate in reactions, significantly improving the generation efficiency of reactive oxygen species and consequently enhancing the antibacterial performance.

Figure S7 presents Tauc plots derived from the Kubelka-Munk transformation, depicting the relationship between (*αhν*)^2^ and photon energy (*hν*). The band gap energies (*E*_*g*_) of TiO_2_ and CoNi-LDH were determined by extrapolating the linear regions of the curves to the abscissa, yielding values of 2.99 eV for TiO_2_ and 2.34 eV for CoNi-LDH. Mott-Schottky plots for TiO_2_ and CoNi-LDH photoelectrodes are displayed in Fig. S8. The flat-band potentials were obtained through linear extrapolation of the potential-dependent capacitance data. The conduction band potentials (*E*_CB_) were subsequently determined as −0.17 V (vs. NHE) for TiO_2_ and -0.58 V (vs. NHE) for CoNi-LDH. The band gap energies (*E*_g_), valence band potentials (*E*_VB_), and *E*_CB_ obtained from these measurements satisfy the fundamental semiconductor relationship:

1 $${E}_{{\rm{g}}}={E}_{{\rm{VB}}}-{E}_{{\rm{CB}}}$$

Based on Eq. (1), the *E*_VB_ were calculated as 2.82 V vs. NHE for TiO_2_ and 1.76 V vs. NHE for CoNi-LDH.

**Fig. 5:**
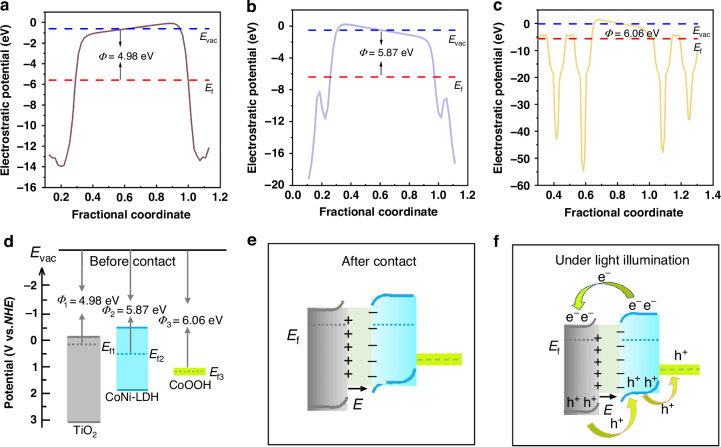
DFT calculations and proposed charge transfer mechanism of TiO_2_, CoNi-LDH, and CoOOH. The results of the work function calculated by DFT **a** TiO_2_, **b** CoNi-LDH, **c** CoOOH. Schematic diagram of band structure and electron transfer of TiO_2_, CoNi-LDH and CoOOH: **d** before contact, **e** after contact, and **f** under light irradiation

Figure 5a–c shows the work functions of TiO_2_, CoNi-LDH, and CoOOH obtained from DFT calculations, with values of 4.98 eV, 5.87 eV, and 6.06 eV, respectively. A smaller work function facilitates electron emission. The relationship among the work function, Fermi level, and vacuum level is as follows:

2 $$\phi={E}_{vac}-{E}_{f}$$

Here, *Ф* represents the work function, *E*_*vac*_ denotes the vacuum level, and *E*_*f*_ corresponds to the Fermi level. Consequently, the relative positions of the Fermi levels for TiO_2_, CoNi-LDH, and CoOOH can be obtained as illustrated in Fig. 5d. Furthermore, based on the results from Figs. S7 and S8, the positions of the conduction band, valence band, and bandgap for TiO₂, CoNi-LDH, and CoOOH are determined and presented in Fig. 5d. When the individual components come into close contact (Fig. 5e), electrons spontaneously transfer from TiO_2_ with a higher Fermi level to CoNi-LDH with a lower Fermi level until their Fermi levels align. This charge redistribution process results in upward band bending at the TiO_2_/CoNi-LDH interface and generates a built‑in electric field directed from TiO_2_ toward CoNi‑LDH. Under illumination, this built‑in electric field acts as a powerful driving force that efficiently injects photogenerated electrons from the conduction band of CoNi‑LDH into the conduction band of TiO_2_, while photogenerated holes in the valence band of TiO_2_ migrate in the opposite direction to the valence band of CoNi‑LDH. The introduction of the CoOOH co‑catalyst further optimizes this process: its valence band position creates an energetic “step” for hole extraction, allowing holes to sequentially transfer from CoNi‑LDH to the surface of CoOOH along the energy ladder, thereby forming a continuous hole‑transfer chain of VB (TiO_2_)→VB (CoNi‑LDH)→CoOOH. This mechanism greatly prolongs the carrier lifetime and suppresses charge recombination.

Figure S9 shows the ESR results of TiO_2_/LDH/−1.7 V, under light irradiation, a distinct 1:2:2:1 quartet signal of the DMPO–·OH adduct was clearly observed in the test system. This directly confirms that the TiO_2_/CoNi-LDH system can generate hydroxyl radicals upon photoexcitation. Simultaneously, the characteristic signal of the DMPO–·O_2_^−^ adduct was also clearly captured for the TiO_2_/CoNi-LDH/−1.7 V composite after light exposure. The experimental results show that the signal intensities of both radicals increased significantly when the irradiation time was extended from 5 min to 10 min, whereas no signals were detected under dark conditions. These direct ESR evidences strongly demonstrate that the TiO_2_/CoNi-LDH heterojunction effectively promotes the separation of photogenerated carriers. However, not all TiO_2_ and CoNi-LDH can form heterojunctions, and in the remaining components that do not form inhibition, the holes in the valence band of TiO_2_ possess sufficient potential to oxidize H_2_O/OH^−^ into ·OH, while the electrons in the conduction band of TiO_2_/CoNi-LDH reduce adsorbed O_2_ to ·O_2_^−^.

**Fig. 6:**
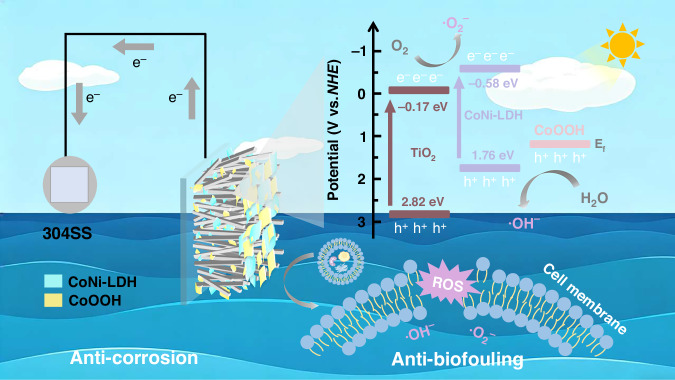
Proposed mechanism of enhanced photocathodic protection and bacterial inactivation. The enhancement mechanism of CoOOH modified TiO_2_/CoNi-LDH heterojunction for photoelectrochemical cathodic protection and bacterial inactivation performance

Figure 6 illustrates the mechanism of the CoOOH-modified TiO_2_/CoNi-LDH heterojunction for PECCP and sterilization. As described in Fig. 5, a type-II heterojunction is formed between TiO_2_ and CoNi-LDH. Driven by the built-in electric field of the heterojunction, the photogenerated electrons and holes on TiO_2_ and CoNi-LDH are spatially separated. This results in the accumulation of photogenerated electrons in the conduction band of TiO_2_, while photogenerated holes accumulate in the valence band of CoNi-LDH. As a cocatalyst, CoOOH facilitates the transfer of photogenerated holes from the valence band of CoNi-LDH to CoOOH and accelerates the corresponding reaction (water oxidation).

Such efficient spatial charge separation directly dictates the dual‑functional performance of the photoanode. In cathodic protection applications, photogenerated electrons accumulated in the conduction band of TiO_2_ travel along the nanorods to the FTO substrate and further migrate to the coupled metal surface, shifting the metal potential negatively into the immune region and thus achieving sustained cathodic polarization protection. Simultaneously, the system exhibits an outstanding synergistic antibacterial effect. The highly oxidizing holes effectively captured by CoOOH, together with reactive oxygen species such as·O_2_^−^ and ·OH generated by the separated carriers reacting with active species in the solution, cooperatively attack the cell membrane and genetic material of *Pseudomonas aeruginosa*, leading to bacterial death.

## Discussion

In this study, a CoOOH-modified TiO_2_/CoNi-LDH heterojunction photoanode was fabricated, demonstrating integrated photoelectrochemical cathodic protection and antibacterial performance. The key finding is that the in situ reconstruction of CoNi-LDH into CoOOH under illumination acts as an efficient co-catalyst, accelerating the transfer/consumption of holes. This reconstructed interface synergizes with the heterojunction, significantly reducing the reaction overpotential and inducing a substantial negative shift of 380 mV in the cathodic potential of 304 SS. Concurrently, the enhanced generation of reactive oxygen species irreversibly damages *Pseudomonas aeruginosa*, achieving complete inactivation within 120 min of illumination.

This dynamically reconstructed heterostructure provides a mechanistic foundation for constructing intelligent protective systems that integrate cathodic protection with broad-spectrum antibacterial capabilities. Although the material exhibits ideal dual-functional characteristics under laboratory evaluation, its long-term chemical stability under coupled multifactor conditions in actual marine service environments, as well as its evolving performance under complex natural sea conditions (such as fluctuating illumination and biofouling), still require further assessment. Future research will focus on conducting field exposure tests in complex marine environments to validate its feasibility for industrial-scale applications.

## Materials and methods

### Materials

All reagents used in the experiments were of analytical grade and used directly. Absolute ethanol (CH_3_CH_2_OH), sodium chloride (NaCl), hydrochloric acid (HCl), and potassium chloride (KCl) were acquired from Sinopharm Chemical Reagent Co., Ltd. Titanium (IV) isopropoxide (C_12_H_28_O_4_Ti), cobalt (II) nitrate hexahydrate (Co(NO_3_)_2_·6H_2_O), and nickel (II) nitrate hexahydrate (Ni(NO_3_)_2_·6H_2_O) were obtained from Macklin Biochemical Technology Co., Ltd. (Shanghai).

### Preparation of TiO_2_ nanorods

First, the FTO conductive glass was cut using a high-precision manual stepping cutter to obtain glass substrates with dimensions of 10 mm × 30 mm × 1.5 mm, which were then ultrasonically treated in anhydrous ethanol and deionized water for 20 min, respectively. A total of 30 mL of HCl was slowly added to 30 mL of deionized water and stirred for 5 min, followed by the addition of 0.3 g of KCl and 720 µL of C_12_H_28_O_4_Ti, with thorough stirring for 40 min. The cleaned FTO conductive glass, with its conductive side facing upward at a certain angle, was placed into a 100 mL high-pressure reaction kettle. The kettle was then placed in an electric blast drying oven at 180 °C for a 6-h reaction. After the hydrothermal reaction, the conductive glass was repeatedly washed with ethanol and deionized water and then dried. The cleaned sample was placed in a muffle furnace for annealing at 450 °C for 2 h, yielding a photoelectrode loaded with TiO_2_ nanorods.

### Preparation of TiO_2_/CoNi-LDH and CoNi-LDH photoanodes

The electrodeposition of CoNi-LDH was performed using a three-electrode system on an electrochemical workstation (VersaSTAT 3 F). An FTO conductive glass with an area of 30 mm × 10 mm served as the working electrode, a R0232 calomel electrode as the reference electrode, and a platinum sheet with an area of 10 mm × 10 mm as the auxiliary electrode. The deposition solution consisted of 20 mM Co(NO_3_)_2_·6H_2_O and 10 mM Ni(NO_3_)_2_·6H_2_O. Constant potential method was employed for electrodeposition, which was conducted at a constant voltage within the cathode potential range of −0.9 to −2.1 V (vs. SCE) for 150 s. After completion of deposition, the samples were rinsed with deionized water and then dried in an oven at 60 °C. The obtained samples were named TiO_2_/CoNi-LDH/−0.9 V, TiO_2_/CoNi-LDH/−1.3 V, TiO_2_/CoNi-LDH/−1.7 V, and TiO_2_/CoNi-LDH/−2.1 V, respectively. For comparison, under the same conditions, direct deposition was carried out on FTO conductive glass without grown TiO_2_ nanorods using Co(NO_3_)_2_·6H_2_O and Ni(NO_3_)_2_·6H_2_O as precursors, which was denoted as CoNi-LDH.

### Characterization

The microstructures of the synthesized materials were characterized using scanning electron microscopy (SEM, Regulus 8100, Hitachi, Japan) and transmission electron microscopy (TEM, JEM-2100PLUS, Japan). Crystal structures were analyzed by X-ray diffraction (XRD, D/MAX-2500/PC, Rigaku, Japan). Elemental composition and chemical states were determined through X-ray photoelectron spectroscopy (XPS, ESCALAB 250Xi, Thermo Fisher Scientific, USA). Material properties were further examined by Raman spectroscopy (DXR2, Thermo Fisher Scientific, USA) and Fourier-transform infrared spectroscopy (FT-IR, Nicolet IS10, Thermo Fisher Scientific, USA). Moreover, Photoluminescence spectroscopy (PL, FLS1000, Edinburgh Instruments, UK) and ultraviolet-visible spectroscopy (UV-Vis DRS, Cary 5000, Agilent Technologies, USA) were employed to further measure the separation ability of photocarriers and optical absorption capacity of the prepared photoanodes.

### Electrochemical and photoelectrochemical measurements

For photogenerated OCP characterization, a R0232 saturated calomel electrode served as the reference electrode (RE) while 304 SS coupled with the tested photoanode functioned as the working electrode (WE). Testing involved five intermittent illumination cycles with 200-s intervals. Photogenerated current density with time curve (*i-t*) measurements required grounding (GND) to prevent polarization interference. The R0232 saturated calomel electrode was maintained as RE with the photoanode as WE, utilizing zero-resistance ammeter (ZRA) mode under identical intermittent illumination conditions (200-s cycles repeated five times).

Electrochemical measurements were conducted in a simulated seawater environment (3.5 wt% NaCl solution) using a three-electrode configuration connected to a VersaSTAT 3 F electrochemical workstation. A Pt electrode and an R0232 saturated calomel electrode served as the counter electrode and reference electrode, respectively, with the fabricated series of photoelectrodes acting as the working electrode.

Linear sweep voltammetry (LSV) curves were acquired under simulated solar illumination (100 mW cm^−2^) by applying a bias potential ranging from −0.5 to 1.5 V, with periodic light on/off cycles every 3 s. Mott-Schottky analysis, performed to determine the semiconductor type and band structure parameters, was carried out by scanning the potential from −1 to 1 V with a potential increment of 0.2 V. The electrochemical impedance spectroscopy (EIS) measurement was conducted using a three-electrode system over a frequency range from 100 kHz to 0.01 Hz. Cyclic voltammetry (CV) measurements were systematically conducted within the non-faradaic potential region at varying scan rates of 10, 30, 50, 70, and 90 mV s^−1^ to obtain the CV profiles for the series of photoelectrodes.

### Evaluation of anti-biofouling properties

All experimental apparatus was sterilized in an autoclave at 120 °C for 30 min. Initially, 1 mL of the bacterial inoculum was added to the prepared nutrient broth, thoroughly mixed, and incubated in a 37 °C constant-temperature shaking incubator for 12 h, the resulting bacterial suspension was designated as the F_0_ generation. An aliquot of equal mass was then transferred from the first-generation (F_0_) suspension into freshly prepared nutrient broth and incubated again under identical conditions (37 °C, shaking, 12 h) to yield the F_1_ generation suspension.

For the photocatalytic reaction, 1 mL of the F_1_ generation suspension and 99 mL of PBS solution were introduced into the photocatalytic reactor, thoroughly mixed, and subsequently combined with the TiO_2_/CoNi-LDH/−1.7 V composite photoelectrode. The entire assembly was positioned beneath a 300 W Xenon lamp equipped with an AM1.5 G filter, adjusted to an illumination intensity of 100 mW cm^−2^. To prevent localized heating from interfering with the results, a recirculating chiller system (bottom-inlet-top-outlet configuration) was connected to the reactor to maintain constant temperature. Prior to illumination, a 100 µL aliquot of the mixed solution was extracted and labeled as *t* = 0 min. The light source was then activated to commence the reaction, with samples collected at 30-min intervals (designated as 30, 60, 90, and 120 min). The collected samples were chronologically labeled. Each sample underwent a 10-fold dilution (dilution factor: 10^1^) by adding 900 µL of deionized water; this dilution step was repeated twice. Subsequently, 100 µL of the thrice-diluted liquid (final dilution factor: 10^3^) was spread onto the surface of Luria Bertani (LB) solid agar plates using the spread plate technique. The inoculated plates were inverted and incubated in a 37 °C constant-temperature incubator, with the corresponding time point marked on the base of each plate. After 24 h of incubation, the plates were removed for colony counting.

### Density functional theory calculations

Surface work functions (*Φ*) were calculated by density functional theory (DFT) in order to evaluate interfacial electron transfer behavior. Geometry optimizations were performed using the CASTEP module with the generalized gradient approximation (GGA) and the Perdew-Burke-Ernzerhof (PBE) functional. Structural relaxation was attained using the conjugate gradient (CG) method, iterated until the total energy error was below 10^−5^ eV, and the force on each atom was less than 0.05 eV/Å. A cutoff energy of 450 eV was applied to the plane-wave basis. The work function was calculated using the DMol3 module. An asymmetric two-layer slab model was constructed, with the top layer relaxed and the bottom layer fixed. A vacuum layer of 20 Å was added along the Z-axis to avoid periodic interactions.

## Supplementary information

Supplemental material

## Data Availability

Data available on request from the authors.
