# Supplementary material for: Light-induced in situ reconstruction of CoOOH-modified TiO2/CoNi-LDH heterojunction photoanode: achieving excellent photoelectrochemical cathodic protection and bacterial inactivation
Source: Light Sci Appl. 2026 May 11;15:230. doi: 10.1038/s41377-026-02328-z (PMC13161232; doi:10.1038/s41377-026-02328-z)
Supplement: Supplementary file 1 — Supplemental material [file 41377_2026_2328_MOESM1_ESM.docx]

**Supporting Information for**

**Light induced in-situ reconstruction of CoOOH modified TiO_2_/CoNi-LDH heterojunction photoanode: achieving excellent photoelectrochemical cathodic protection and bacterial inactivation**

Meiqi Wang, Yiqing Tang, Juan Liu, Xinyue Feng, Zheng Kuang, Yingnan Qin, Jing Tian^*^, Ning Wang^*^, Jing Wang^[[1]](#footnote-1)^

College of Chemical Engineering, Qingdao University of Science and Technology, 53 Zhengzhou Road, Qingdao 266042, Shandong, China

**Fig. S1** Optical micrographs of 304 stainless steel prior to and following a 24-hour immersion in a 3.5 wt.% NaCl solution with PECCP by TiO_2_/CoNi-LDH/−1.7 V.

**Fig. S2** Optical micrographs of 304 stainless steel prior to and following a 24-hour immersion in a 3.5 wt.% NaCl solution without PECCP.

**Fig. S3** Photographs of Pseudomonas aeruginosa colony plates subjected to different durations of light exposure under control conditions.

**Fig. S4** EDS diagram of TiO_2_/CoNi-LDH/−1.7 V.

**Fig. S5** Element mapping of TiO_2_/CoNi-LDH/−1.7 V.

**Fig. S6** Cyclic voltammetry curves of photoelectrodes (A)TiO_2_, (B)LDH, (C)TiO_2_/LDH/−1.7 V, and (D)TiO_2_/LDH/−1.7 V after.

**Fig. S7** the bandgap width of TiO_2_ and CoNi-LDH.

**Fig. S8** Mott-schottky plots of (A)TiO_2_, (B)CoNi-LDH.

**Fig. S9** ESR spectra of TiO_2_/LDH/−1.7 V under different illumination conditions (A)·OH , (B)·O_2_^−^

1. Corresponding author

   E-mail address: [tianjing@qust.edu.cn](mailto:tianjing@qust.edu.cn) (Jing Tian); [04031@qust.edu.cn](mailto:04031@qust.edu.cn) (Ning Wang); w[angjing04032@qust.edu.cn](mailto:angjing04032@qust.edu.cn) (Jing Wang) [↑](#footnote-ref-1)
